# Assessment of US Preventive Services Task Force Guideline–Concordant Cervical Cancer Screening Rates and Reasons for Underscreening by Age, Race and Ethnicity, Sexual Orientation, Rurality, and Insurance, 2005 to 2019

**DOI:** 10.1001/jamanetworkopen.2021.43582

**Published:** 2022-01-18

**Authors:** Ryan Suk, Young-Rock Hong, Suja S. Rajan, Zhigang Xie, Yenan Zhu, Jennifer C. Spencer

**Affiliations:** 1 Department of Management, Policy, and Community Health, University of Texas Health Science Center at Houston School of Public Health, Houston; 2 Department of Health Services Research, Management and Policy, College of Public Health and Health Professions, University of Florida, Gainesville; 3 University of Florida Health Cancer Center, Gainesville; 4 Department of Population Health, Dell Medical School, University of Texas at Austin, Austin; 5 Department of Internal Medicine, Dell Medical School, University of Texas at Austin, Austin

## Abstract

**Importance:**

Cervical cancer screening rates are suboptimal in the US. Population-based assessment of reasons for not receiving screening is needed, particularly among women from historically underserved demographic groups.

**Objective:**

To estimate changes in US Preventive Service Task Force guideline–concordant cervical cancer screening over time and assess the reasons women do not receive up-to-date screening by sociodemographic factors.

**Design, Setting, and Participants:**

This pooled population-based cross-sectional study used data from the US National Health Interview Survey from 2005 and 2019. A total of 20 557 women (weighted, 113.1 million women) aged 21 to 65 years without previous hysterectomy were included. Analyses were conducted from March 30 to August 19, 2021.

**Exposures:**

Sociodemographic factors, including age, race and ethnicity, sexual orientation, rurality of residence, and health insurance type.

**Main Outcomes and Measures:**

Primary outcomes were US Preventive Services Task Force guideline–concordant cervical cancer screening rates and self-reported primary reasons for not receiving up-to-date screening. For 2005, up-to-date screening was defined as screening every 3 years for women aged 21 to 65 years. For 2019, up-to-date screening was defined as screening every 3 years with a Papanicolaou test alone for women aged 21 to 29 years and screening every 3 years with a Papanicolaou test alone or every 5 years with high-risk human papillomavirus testing or cotesting for women aged 30 to 65 years. Population estimation included sampling weights.

**Results:**

Among 20 557 women (weighted, 113.1 million women) included in the study, most were aged 30 to 65 years (16 219 women; weighted, 86.3 million women [76.3%]) and had private insurance (13 571 women; weighted, 75.8 million women [67.0%]). With regard to race and ethnicity, 997 women (weighted, 6.9 million women [6.1%]) were Asian, 3821 women (weighted, 19.5 million women [17.2%]) were Hispanic, 2862 women (weighted, 14.8 million women [13.1%]) were non-Hispanic Black, 12 423 women (weighted, 69.0 million women [61.0%]) were non-Hispanic White, and 453 women (weighted, 3.0 million women [2.7%]) were of other races and/or ethnicities (including Alaska Native and American Indian [weighted, 955 000 women (0.8%)] and other single and multiple races or ethnicities [weighted, 2.0 million women (1.8%)]). In 2019, women aged 21 to 29 years had a significantly higher rate of overdue screening (29.1%) vs women aged 30 to 65 years (21.1%; *P* < .001). In both age groups, the proportion of women without up-to-date screening increased significantly from 2005 to 2019 (from 14.4% to 23.0%; *P* < .001). Significantly higher rates of overdue screening were found among those of Asian vs non-Hispanic White race and ethnicity (31.4% vs 20.1%; *P* = .01), those identifying as LGBQ+ (gender identity was not assessed because of a small sample) vs heterosexual (32.0% vs 22.2%; *P* < .001), those living in rural vs urban areas (26.2% vs 22.6%; *P* = .04), and those without insurance vs those with private insurance (41.7% vs 18.1%; *P* < .001). The most common reason for not receiving timely screening across all groups was lack of knowledge, ranging from 47.2% of women identifying as LGBQ+ to 64.4% of women with Hispanic ethnicity. Previous receipt of a human papillomavirus vaccine was not a primary reason for not having up-to-date screening (<1% of responses). From 2005 to 2019, among women aged 30 to 65 years, lack of access decreased significantly as a primary reason for not receiving screening (from 21.8% to 9.7%), whereas lack of knowledge (from 45.2% to 54.8%) and not receiving recommendations from health care professionals (from 5.9% to 12.0%) increased significantly.

**Conclusions and Relevance:**

This cross-sectional study found that cervical cancer screening that was concordant with US Preventive Services Task Force guidelines decreased in the US between 2005 and 2019, with lack of knowledge reported as the biggest barrier to receiving timely screening. Campaigns addressing patient knowledge and provider communication may help to improve screening rates, and cultural adaptation of interventions is needed to reduce existing disparities.

## Key Points

**Question:**

What proportion of screening-eligible women do not have up-to-date US Preventive Services Task Force guideline–concordant cervical cancer screening status, and what are their primary reasons for not receiving timely screening?

**Findings:**

In this cross-sectional study of 20 557 women (weighted, 113 million women) eligible for cervical cancer screening in the US, the proportion of women without up-to-date screening significantly increased from 14.4% in 2005 to 23.0% in 2019 among all sociodemographic groups, with disparities found across different sociodemographic groups and lack of knowledge reported as the biggest barrier to receiving screening.

**Meaning:**

This study found that guideline-concordant cervical cancer screening rates decreased between 2005 and 2019; campaigns addressing patient knowledge and practitioner communication may help to improve cervical cancer screening rates, and cultural adaptation of interventions is needed to reduce existing disparities.

## Teaser

This cross-sectional study assesses the rates of US Preventive Services Task Force guideline–concordant cervical cancer screening in the US from 2005 to 2019 and examines the sociodemographic factors associated with women’s self-reported reasons for not receiving up-to-date screening.

## Introduction

Cervical cancer screening has substantially reduced the incidence and mortality of cervical cancer in the US over the past 40 years,^1^ and this screening consistently receives an A rating, the highest level of recommendation offered by the US Preventive Services Task Force (USPSTF). Current recommendations suggest that women aged 21 to 29 years with average risk receive cytological screening every 3 years, with screening intervals for those aged 30 to 65 years extended to every 5 years if receiving human papillomavirus (HPV) testing alone or HPV cotesting with cytological screening.^2^ Healthy People 2030 estimated in 2018 that the proportion of women who were up to date on cervical cancer screening was 80.5%, with a target rate of 84.3%.^3^ However, there is substantial variation in cervical cancer screening rates by race and/or ethnicity, rurality of residence, insurance type, and sexual orientation,^4^ and multiple studies and national reports have reported that the national rate of guideline-concordant screening has been decreasing over the past 5 years.^5,6,7,8,9^ Watson et al^5,6^ found that this decrease has been more pronounced among younger women (aged 21-29 years), although the factors associated with these changes are unknown.

To improve cervical cancer prevention in the US, it is important to identify potentially modifiable reasons underlying the low and decreasing rates of cervical cancer screening, particularly among traditionally underserved populations (eg, racial and ethnic minority groups and those identifying as LGBQ+ [we could not identify transgender individuals in the data, which include only a binary sex variable defined as male or female; hence, we used the term *LGBQ+*]). Previous studies assessed knowledge and attitudes toward cervical cancer screening among racial and ethnic minority women, finding knowledge of HPV and cervical cancer screening may be lower among Asian, Black, and Hispanic women.^10,11,12,13^ To improve population-level cervical cancer screening rates, population-based assessment of the most common reasons for underscreening, particularly among historically underserved populations, is needed.

We aimed to estimate recent patterns in cervical cancer screening use and the primary reasons for not receiving up-to-date screening by age, race and ethnicity, sexual orientation, rurality of residence, and insurance type using a nationally representative survey. We also aimed to examine the proportions of women who did not receive up-to-date screening and the reasons for not receiving screening in 2005 compared with 2019 to assess whether these proportions and reasons have changed significantly over 14 years.

## Methods

### Data Source

In this pooled cross-sectional study, we used 2005 and 2019 data from the National Health Interview Survey (NHIS) of the National Center for Health Statistics of the Centers for Disease Control and Prevention. Analyses were conducted from March 30 to August 19, 2021. The NHIS is a cross-sectional household survey representing noninstitutionalized civilians residing in the US. We used the sample adult file, which includes individuals randomly selected from participating households who were asked about a variety of demographic, health behavior, and health care characteristics in a structured face-to-face interview. Complete details on NHIS methodology are available online.^14^ This study was approved by the University of Texas Health Science Center and deemed exempt from additional institutional review because it involved analysis of publicly available and deidentified data. This study followed the Strengthening the Reporting of Observational Studies in Epidemiology (STROBE) reporting guideline for cross-sectional studies.

### Measures

Our primary outcome was being overdue for screening per the USPSTF-recommended cervical cancer screening schedule (ie, not up to date). For 2005, guideline-concordant up-to-date screening was defined as screening every 3 years for women aged 21 to 65 years based on USPSTF guidelines and clinical recommendations. For 2019, up-to-date screening was defined as screening every 3 years with a Papanicolaou test alone among women aged 21 to 29 years and screening every 3 years with a Papanicolaou test alone or every 5 years when conducting high-risk HPV testing or cotesting among women aged 30 to 65 years.^2^ We assessed age, time since the most recent screening, and HPV DNA testing status to identify whether the participant had up-to-date screening.^2^ We excluded those reporting a previous hysterectomy from the study sample because the reasons for hysterectomy were not assessed; therefore, we could not ascertain whether or how frequently cervical cancer screening was indicated for these participants.

We also examined the reasons for not receiving cervical cancer screening (ever or for the last 3 or 5 years) among those who were not up to date with the USPSTF-recommended screening schedule. The NHIS asks participants who reported that they have never received screening for more than 3 or 5 years the following questions: (1) “What is the main reason that you have not had a cervical cancer screening ever or in the last 3 years?” (for 2005 survey) or (2) “What is the main reason that you have not had a cervical cancer screening ever or in the last 5 years?” (for 2019 survey). The participants selected only one of the following 10 answers: (1) never thought about it; (2) didn’t know I needed this type of test; (3) doctor didn’t say I needed it; (4) haven’t had any problems; (5) put it off; (6) too expensive/no insurance; (7) too painful, unpleasant, or embarrassing; (8) don’t have a doctor; (9) had HPV vaccine (2019 survey only); and (10) other. We combined similar answers for the analysis; responses of “never thought about it” and “didn’t know I needed this type of test” were combined into “didn’t know I needed it,” and responses of “too expensive/no insurance” and “don’t have a doctor” were combined into “lack of access.”

Independent variables included in the study were age (categorical as 21-29 years or 30-65 years), race and ethnicity (Asian, Hispanic, non-Hispanic Black, non-Hispanic White, and other race or ethnicity [including Alaska Native, American Indian, and other single and multiple races or ethnicities]), rurality of residence (rural vs urban; nonmetropolitan counties were designated as rural, and metropolitan counties [small, medium, large central, and large fringe] were designated as urban), and insurance type (private, public, other insurance, and no insurance). We also assessed whether sexual orientation was associated with screening status; for this, we combined all orientations other than heterosexual (gay, lesbian, bisexual, other, and unsure) into a single LGBQ+ category due to the small sample size. We could not identify transgender individuals in the NHIS data, which includes only a binary sex variable defined as male or female.

### Statistical Analysis

We first generated descriptive statistics to summarize the distribution of the independent variables for the sample population. We then estimated the prevalence of each outcome and calculated 95% CIs and *P* values for each of the independent variables. We used a Wald test adjusted for survey design for the bivariate analyses describing overdue cervical cancer screening status and the primary reasons for not receiving timely screening. We compared the outcomes across sociodemographic factors in 2019 and assessed the outcomes in 2005 compared with 2019 by age group, race and ethnicity, and insurance type. We could not compare by year for sexual orientation and rurality of residence because of the lack of data on these variables in 2005. We also estimated the predicted probabilities of not having up-to-date screening by age group using multivariable logistic regression models, simultaneously adjusting for sociodemographic factors. The outcome of this process can be interpreted as a representation of the direct residual effect of each demographic characteristic (ie, the expected probability of not having up-to-date screening for a counterfactual population that is identical to the full sample in all demographic characteristics with the exception of the single independent variable being examined). The proc survey procedures in SAS software, version 9.4 (SAS Institute Inc), were used for population estimation, which included weight, cluster, and strata statements and incorporated sampling weights. Statistical significance was set at *P* < .05, and all analyses were performed using SAS software, version 9.4.

## Results

Among 20 557 women (weighted, 113.1 million women) included in the study, most were aged 30 to 65 years (16 219 women; weighted, 86.3 million women [76.3%]) and had private insurance (13 571 women; weighted, 75.8 million women [67.0%]). With regard to race and ethnicity, 997 women (weighted, 6.9 million women [6.1%]) were Asian, 3821 women (weighted, 19.5 million women [17.2%]) were Hispanic, 2862 women (weighted, 14.8 million women [13.1%]) were non-Hispanic Black, 12 423 women (weighted, 69.0 million women [61.0%]) were non-Hispanic White, and 453 women (weighted, 3.0 million women [2.7%]) were of other races and/or ethnicities (including Alaska Native and American Indian [weighted, 955 000 women (0.8%)] and other single and multiple races or ethnicities [weighted, 2.0 million women (1.8%)]).

In 2005, among 10 668 women (weighted, 31.8 million women) eligible for screening, 2466 women (weighted, 7.6 million women [23.9%]) were aged 21 to 29 years, and 6901 women (weighted, 21.9 million women [69.2%]) had private insurance (Table 1). With regard to race and ethnicity, 338 women (weighted, 1.1 million women [3.5%]) were Asian, 2188 women (weighted, 3.9 million women [12.2%]) were Hispanic, 1632 women (weighted, 4.2 million women [13.3%]) were non-Hispanic Black, 6345 women (weighted, 22.0 million women [69.4%]) were non-Hispanic White, and 165 women (weighted, 538 000 women [1.7%]) were of other races and/or ethnicities (including Alaska Native and American Indian [weighted, 168 000 women (0.5%)] and other single and multiple races or ethnicities [weighted, 370 000 women (1.2%)]). A total of 1600 women (weighted, 4.5 million women [14.4%; 95% CI, 13.7%-15.2%]) did not have up-to-date screening.

**Table 1.** Participant Baseline Characteristics, 2005 and 2019^a^

Characteristic	2005		2019	
	Unweighted, No.	Weighted, No. (%) [95% CI]	Unweighted, No.	Weighted, No. (%) [95% CI]
Total participants, No.	10 668	31 760 368	9889	81 330 124
Screening status^b^				
Not up to date	1600	4 507 734 (14.4) [13.7-15.2]	2049	18 256 280 (23.0) [21.9-24.1]
Up to date	8894	26 760 575 (85.6) [84.8-86.3]	7599	61 083 789 (77.0) [75.9-78.1]
Age range, y				
21-29	2466	7 576 919 (23.9) [22.8-24.9]	1872	19 248 536 (23.7) [22.6-24.8]
30-65	8202	24 183 449 (76.1) [75.1-77.2]	8017	62 081 588 (76.3) [75.2-77.4]
Race and ethnicity				
Asian	338	1 097 489 (3.5) [3.0-3.9]	659	5 775 864 (7.1) [6.4-7.8]
Hispanic	2188	3 860 886 (12.2) [11.4-13.0]	1633	15 640 028 (19.2) [17.7-20.8]
Non-Hispanic Black	1632	4 215 162 (13.3) [12.4-14.2]	1231	10 537 421 (13.0) [11.8-14.1]
Non-Hispanic White	6345	22 049 055 (69.4) [68.1-70.7]	6078	46 953 174 (57.7) [55.9-59.6]
Other^c^	165	537 776 (1.7) [1.4-2.0]	288	2 423 638 (3.0) [2.2-3.8]
Sexual orientation^d^				
Heterosexual	NA	NA	9141	74 795 904 (92.8) [92.2-93.5]
LGBQ+^e^	NA	NA	651	5 765 315 (7.2) [6.5-7.9]
Area of residence^d^	NA	NA		
Rural	NA	NA	1309	9 981 430 (12.3) [11.1-13.5]
Urban	NA	NA	8580	71 348 694 (87.7) [86.6-88.9]
Insurance type				
Private	6901	21 911 120 (69.2) [68.0-70.4]	6670	53 932 420 (67.6) [66.1-69.0]
Public	1231	3 131 127 (9.9) [9.2-10.5]	1445	12 456 674 (15.6) [14.5-16.7]
Other	415	1 237 995 (3.9) [3.4-4.4]	415	2 810 915 (3.5) [3.0-4.0]
None	2089	5 390 994 (17.0) [16.2-17.9]	1113	10 628 194 (13.3) [12.3-14.4]

Abbreviations: LGBQ+, lesbian, gay, bisexual, other, and unsure; NA, not applicable or not available.

^a^ Based on nonmissing data for cervical cancer screening behavior from the National Health Interview Survey.

^b^ Screening data were missing for 415 participants (weighted, 2 482 114), including 61 women (weighted, 369 598) aged 21 to 29 years and 354 women (weighted, 2 112 516) aged 30 to 65 years.

^c^ Other category includes Alaska Native and American Indian (weighted, 168 259 women [0.5%] in 2005 and 787 000 women [1.0%] in 2019) and other single and multiple races or ethnicities (weighted, 369 517 women [1.2%] in 2005 and 1 636 638 women [2.0%] in 2019).

^d^ Data were not available for 2005.

^e^ All sexual orientations other than heterosexual (gay, lesbian, bisexual, other sexual orientation, and/or unsure about sexual orientation) were combined into a single LGBQ+ category because of the small sample size; transgender individuals could not be identified in the data, which include only a binary sex variable defined as male or female; hence the use of the term *LGBQ+*.

In 2019, of 9889 women (weighted, 81.3 million women) eligible for screening, 1872 women (weighted, 19.2 million women [23.7%]) were aged 21 to 29 years, 9141 women (weighted, 74.8 million women [92.8%]) were heterosexual, 8580 women (weighted, 71.3 million women [87.7%)] resided in urban areas, and 6670 women (weighted, 53.9 million women [67.6%]) had private insurance (Table 1). With regard to race and ethnicity, 659 women (weighted, 5.8 million women [7.1%]) were Asian, 1633 women (weighted, 15.6 million women [19.2%]) were Hispanic, 1231 women (weighted, 10.5 million women [13.0%]) were non-Hispanic Black, 6078 women (weighted, 47.0 million women [57.7%]) were non-Hispanic White, and 288 women (weighted, 2.4 million women [3.0%]) were of other races and/or ethnicities (including Alaska Native and American Indian [weighted, 787 000 (1.0%)] and other single and multiple races or ethnicities [weighted, 1.5 million (2.0%)]). A total of 2049 women (weighted, 18.3 million women [23.0%; 95% CI, 21.9%-24.1%]; *P* < .001 compared with 14.4% of women in 2005) were not up to date with recommended cervical cancer screening.

### Rates and Reasons for Overdue Screening by Age Over Time

**Figure 1.**
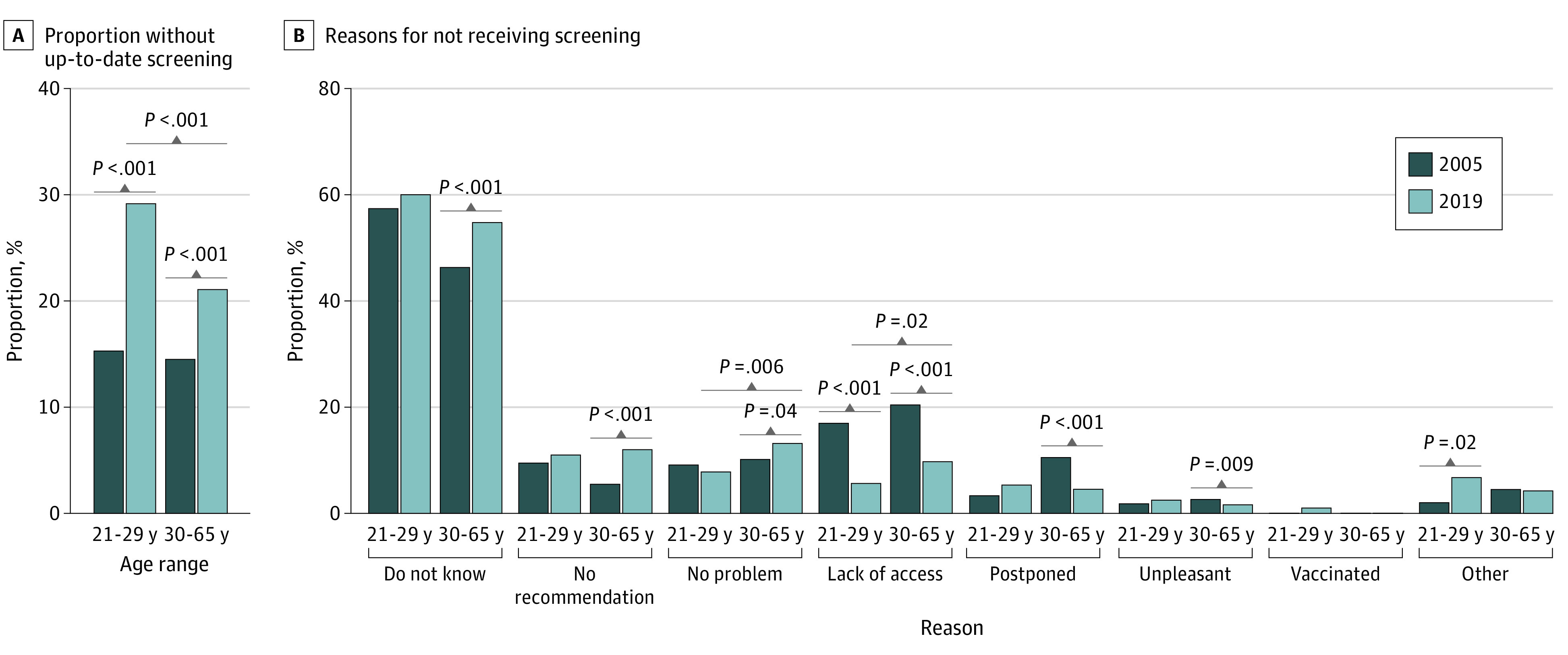
Proportion of Individuals Without Up-to-date Screening and Primary Reasons For Not Receiving Screening by Age Group, 2005 vs 2019

When we stratified by age, we found that the rates of overdue screening and the reasons for not receiving screening differed significantly by age (Figure 1; eTable 1 and eTable 2 in the Supplement). A higher proportion of women aged 21 to 29 years (481 women; weighted, 5.5 million women [29.1%; 95% CI, 26.4%-31.9%]) did not have up-to-date screening compared with women aged 30 to 65 years (1568 women; weighted, 12.7 million women [21.1%; 95% CI, 19.9%-22.3%]; *P* < .001). For both age groups, not knowing they needed screening was the most common reason for not having up-to-date screening (aged 21-29 years: 257 women [weighted, 3.0 million women (60.0%; 95% CI, 54.6%-65.5%)]; aged 30-65 years: 691 women [weighted, 5.9 million women (54.8%; 51.4%-58.2%)]). Compared with women aged 21 to 29 years, a significantly higher proportion of those aged 30 to 65 years reported they did not receive screening primarily because they did not have any problems (175 women [weighted, 1.4 million women (13.2%; 95% CI, 10.7%-15.6%)] vs 38 women [weighted, 391 000 women (7.8%; 95% CI, 4.8%-10.7%)]; *P* = .006) or did not have access to screening (121 women [weighted, 1.0 million women (9.7%; 95% CI, 7.9%-11.6%)] vs 21 women [weighted, 283 000 women (5.6%; 95% CI, 2.8%-8.5%)]; *P* = .02). Previous receipt of an HPV vaccine was not a primary reason for not having up-to-date screening (<1% of total responses [1.0% among women aged 21-29 years and 0% among women aged 30-65 years]).

When we compared the results between 2005 and 2019, the proportion of women who were overdue for screening significantly increased over time in both younger and older groups (eTable 3 and eTable 4 in the Supplement). In 2005, the proportion of women aged 21 to 29 years without up-to-date screening was 14.2% (weighted, 2.5 million women), and this proportion almost doubled to 29.1% by 2019 (weighted, 5.5 million women; *P* < .001). The proportion without up-to-date screening also significantly increased among women aged 30 to 65 years, from 14.5% (weighted, 3.4 million women) in 2005 to 21.1% (weighted, 12.7 million women; *P* < .001) in 2019. When we compared the change in primary reasons for not receiving screening over time among women aged 21 to 29 years, the proportion of women responding that they lacked access to screening decreased significantly (62 women [weighted, 183 800 women (17.3%; 95% CI, 12.6%-22.0%)] in 2005 vs 21 women [weighted, 283 000 women (5.6%; 95% CI, 2.8%-8.5%)] in 2019; *P* < .001), whereas the proportion who had other reasons increased significantly (8 women [weighted, 24 000 women (2.3%; 95% CI, 0.5%-4.1%)] in 2005 vs 26 women [weighted, 340 000 women (6.8%; 95% CI, 3.6%-9.9%)] in 2019; *P* = .02). When comparing the change in primary reasons for not receiving screening over time among women aged 30 to 65 years, the proportion of women responding that they lacked access to screening decreased significantly (271 women [weighted, 750 000 women (21.8%; 95% CI, 19.2%-24.3%)] in 2005 vs 121 women [weighted, 1.0 million women (9.7%; 95% CI, 7.9%-11.6%)] in 2019; *P* < .001), as did the proportions responding that they had postponed screening (115 women [weighted, 333 000 women (9.7%; 95% CI, 7.9%-11.5%)] in 2005 vs 66 women [weighted, 484 000 women (4.5%; 95% CI, 3.3%-5.7%)] in 2019; *P* < .001) or found screening unpleasant (39 women [weighted, 108 000 women (3.1%; 95% CI, 2.2%-4.1%)] in 2005 vs 30 women [weighted, 174 000 women (1.6%; 95% CI, 1.0%-2.3%)] in 2019; *P* = .009), whereas those responding that they did not know they needed screening (563 women [weighted, 1.6 million women (45.2%; 95% CI, 42.3%-48.1%)] in 2005 vs 691 women [weighted, 5.9 million women (54.8%; 95% CI, 51.4%-58.2%)] in 2019; *P* < .001) or they did not receive a recommendation from a health care professional (71 women [weighted, 202 000 women (5.9%; 95% CI, 4.5%-7.2%)] in 2005 vs 162 women [weighted, 1.3 million women (12.0%; 95% CI, 10.0%-14.0%)] in 2019; *P* < .001) significantly increased.

### Rates and Reasons for Overdue Screening by Sociodemographic Factors

**Figure 2.**
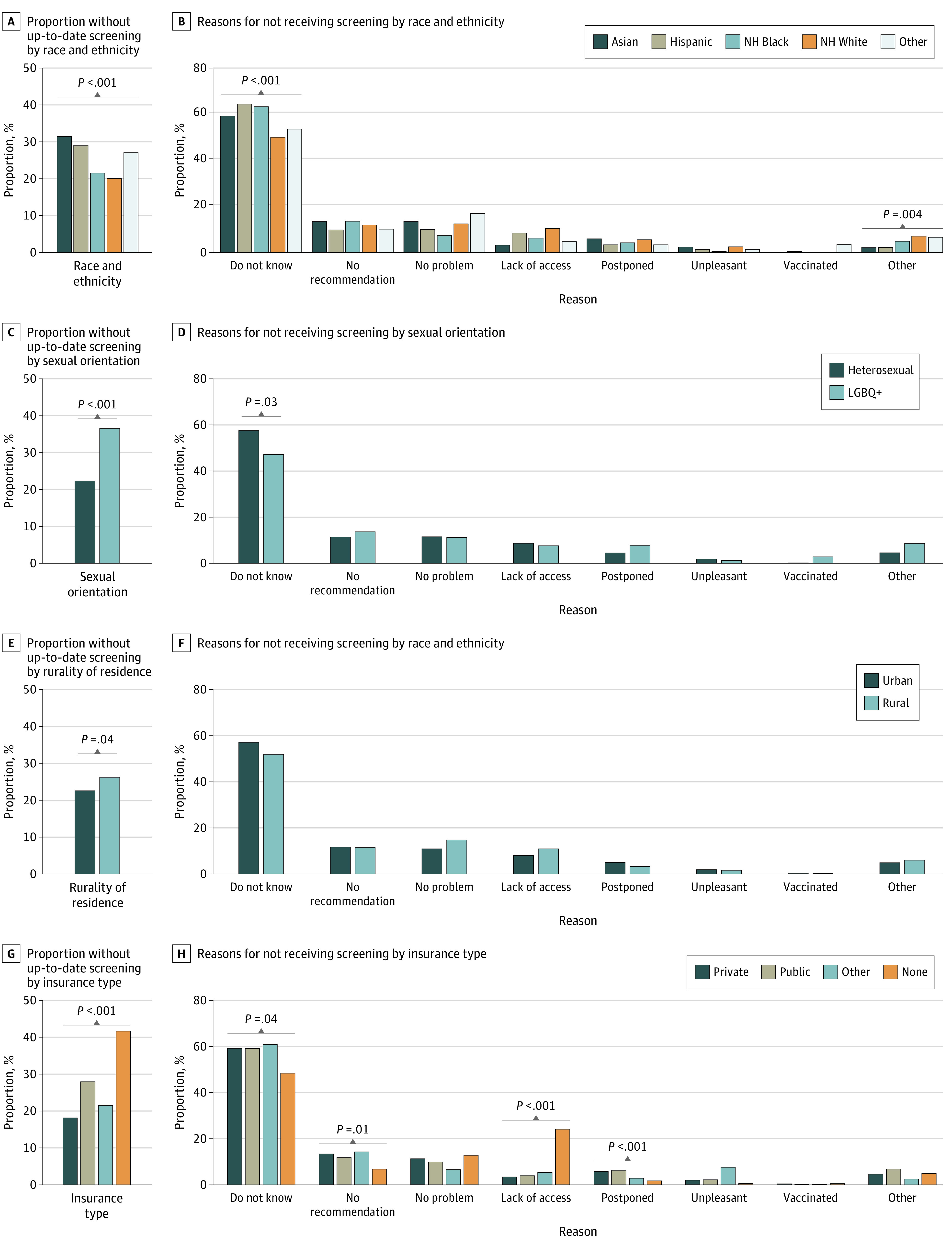
Proportion of Individuals Without Up-to-date Screening and Primary Reasons for Not Receiving Screening by Race and Ethnicity, Sexual Orientation, Rurality of Residence, and Insurance Type, 2019 NH indicates non-Hispanic; and LGBQ+, lesbian, gay, bisexual, other, and/or unsure.

We assessed the rates of overdue screening and the reasons for not receiving screening by other sociodemographic factors, and we also found variation across the groups (Figure 2; eTable 1 and eTable 2 in the Supplement). By race and ethnicity, we found that non-Hispanic White women had the lowest rate of overdue screening (1116 women; weighted, 9.2 million women [20.1%; 95% CI, 18.8%-21.4%]), and Asian women had the highest rate of overdue screening (186 women; weighted, 1.8 million women [31.4%; 95% CI, 27.0%-35.8%]). Across racial and ethnic groups, a significantly higher proportion of Hispanic women reported they did not receive screening because they did not know they needed it (247 women; weighted, 2.6 million women [64.4%, 95% CI, 58.9%-69.9%]), whereas non-Hispanic White women were least likely to answer the same (442 women; weighted, 3.8 million women [50.0%, 95% CI, 45.7%-54.4%]). However, non-Hispanic White women were the most likely to report they did not receive screening primarily because they did not have access to it (90 women; weighted, 785 000 women [10.4%, 95% CI, 8.0%-12.8%]), whereas only a small proportion of Asian women answered the same (7 women; weighted, 53 000 women [3.2%; 95% CI, 0.6%-5.8%]).

When we compared rates and reasons across sexual orientation, a significantly higher proportion of women identifying as LGBQ+ were not up to date in screening vs women identifying as heterosexual (185 women [weighted, 1.8 million women (32.0%; 95% CI, 27.5%-36.5%)] vs 1838 women [weighted, 16.2 million women (22.2%; 95% CI, 21.1%-23.4%)]; *P* < .001) (eTable 1 in the Supplement). Among those without up-to-date screening, a significantly higher percentage of women identifying as heterosexual vs LGBQ+ reported they did not receive screening primarily because they did not know they needed it (862 women [weighted, 8.0 million women (57.5%; 95% CI, 54.5%-60.6%)] vs 75 women [weighted, 779 000 women (47.2%; 95% CI, 37.9%-56.6%)]; *P* = .03). We found that those living in rural areas had a significantly higher rate of overdue screening than those living in urban areas (325 women [weighted, 2.5 million women (26.2%; 95% CI, 22.8%-29.6%)] vs 1724 women [weighted, 15.7 million women (22.6%; 95% CI, 21.4%-23.8%)]; *P* = .04). However, we did not find any significant differences in reasons for not receiving screening between these 2 groups. A significantly higher proportion of women without insurance were not up to date in screening (434 women; weighted, 4.3 million women [41.7%; 95% CI, 38.1%-45.2%]) compared with women with private insurance (1087 women; weighted, 9.6 million women [18.1%; 95% CI, 16.9%-19.3%]).

Across insurance groups, women without up-to-date screening who had other insurance (ie, not public or private) were most likely to report they did not know they needed it (37 women; weighted, 306 900 women [60.8%; 95% CI, 46.9%-74.8%]), whereas women who had no insurance were the least likely to report the same (181 women; weighted 1.8 million women [48.6%; 95% CI, 42.3%-55.0%]) (eTable 2 in the Supplement). Women with private insurance were most likely to report they did not receive screening because they did not have a recommendation from a health care professional (123 women; weighted, 1.1 million women [13.4%; 95% CI, 10.7%-16.1%]), whereas women without insurance were the least likely to report the same (24 women; weighted, 255 000 women [6.8%; 95% CI, 3.8%-9.8%]). However, women without insurance reported that they did not receive screening because of lack of access approximately 7 times more often than women with private insurance (94 women [weighted, 904 000 women (24.1%; 95% CI, 19.1%-29.1%)] vs 30 women [weighted, 275 000 women (3.4%; 95% CI, 2.0%-4.8%)]).

We also examined 2005 compared with 2019 by race and ethnicity and insurance status (eTable 3 and eTable 4 in the Supplement). When comparing over time by race and ethnicity, we found that all racial and ethnic groups, with the exception of Asian women, had a significant increase in the proportion of individuals without up-to-date screening. Although the proportion did not increase over time, Asian women continued to represent the highest proportion of individuals without up-to-date screening (104 women [weighted, 342 000 women (32.5%; 95% CI, 26.6%-38.3%)] in 2005 vs 186 women [weighted, 1.8 million women (31.4%; 95% CI, 27.0%-35.8%)] in 2019; *P* = .78). The proportion of women responding “did not know” as the primary reason for not receiving timely screening significantly increased across all racial and ethnic groups, with the exception of Asian women (66 women [weighted, 219 000 women (63.9%; 95% CI, 54.0%-73.7%)] in 2005 vs 101 women [weighted, 982 000 women (59.2%; 95% CI, 50.0%-68.4%)] in 2019; *P* = .50). The proportion of women without up-to-date screening significantly increased across all insurance types in 2019, whereas women without insurance continued to represent the highest proportion without up-to-date screening (637 women [weighted, 1.6 million women (30.9%; 95% CI, 28.6%-33.1%)] in 2005 vs 434 women [weighted, 4.3 million women (41.7%; 95% CI, 38.1%-45.2%)] in 2019; *P* < .001). Among women without insurance in 2019, significantly fewer reported they did not receive screening primarily because they did not have access (250 women [weighted, 683 000 women (41.8%; 95% CI, 37.3%-46.3%)] in 2005 vs 94 women [weighted, 904 000 women (24.1%; 95% CI, 19.1%-29.1%)] in 2019; *P* < .001). However, significantly more women without insurance in 2019 responded that they did not receive screening primarily because they did not know they needed it (254 women [weighted, 606 000 women (37.1%; 95% CI, 33.1%-41.1%)] in 2005 vs 181 women [weighted, 824 000 women (48.6%; 95% CI, 42.3%-55.0%)] in 2019; *P* = .003).

### Predicted Probabilities of Having Overdue Screening

We simultaneously adjusted for sociodemographic factors to estimate the predicted probabilities of being overdue for recommended screening by age group when holding all other demographic characteristics fixed (Table 2). Among women aged 21 to 29 years, those of Asian race (predicted probability, 36.0%; 95% CI, 34.9%-37.1%), those living in rural areas (predicted probability, 27.9%; 95% CI, 26.1%-29.7%), those without insurance (predicted probability, 41.2%; 95% CI, 40.4%-42.0%), and those who identified as LGBQ+ (predicted probability, 32.4%; 95% CI, 30.9%-33.9%) had the highest adjusted probabilities of having screening that was not up to date. The results were similar among those in the older age group. For women aged 30 to 65 years, the adjusted probability of not having up-to-date screening was highest among those of Asian race (predicted probability, 36.3%; 95% CI, 35.5%-37.1%), those living in rural areas (predicted probability, 24.9%; 95% CI, 23.8%-26.0%), those without insurance (predicted probability, 40.9%; 95% CI, 40.3%-41.6%), and those who identified as LGBQ+ (predicted probability, 32.3%; 95% CI, 30.4%-34.1%).

**Table 2.** Adjusted Predicted Probabilities of Overdue Cervical Cancer Screening, 2019^a^

Variable	Probability of having overdue screening, % (95% CI)^b^			
	Age 21-29 y		Age 30-65 y	
	Predicted	Difference^c^	Predicted	Difference^c^
Race and ethnicity				
Asian	36.0 (34.9 to 37.1)	14.1 (12.9 to 15.4)	36.3 (35.5 to 37.1)	15.8 (14.9 to 16.6)
Hispanic	30.9 (29.7 to 32.1)	9.1 (7.7 to 10.4)	30.1 (29.3 to 31.0)	9.6 (8.7 to 10.5)
Non-Hispanic Black	23.0 (21.8 to 24.2)	1.1 (−0.2 to 2.5)	21.2 (20.6 to 21.7)	0.6 (0.1 to 1.2)
Non-Hispanic White	21.8 (21.3 to 22.4)	1 [Reference]	20.5 (20.3 to 20.8)	1 [Reference]
Other^d^	28.4 (26.1 to 30.7)	6.5 (4.2 to 8.8)	29.3 (26.7 to 31.9)	8.8 (6.2 to 11.4)
Sexual orientation				
Heterosexual	24.1 (23.6 to 24.6)	1 [Reference]	23.3 (23.0 to 23.6)	1 [Reference]
LGBQ+^e^	32.4 (30.9 to 33.9)	8.3 (6.7 to 9.9)	32.3 (30.4 to 34.1)	8.9 (7.0 to 10.8)
Area of residence				
Rural	27.9 (26.1 to 29.7)	3.1 (1.2 to 5.0)	24.9 (23.8 to 26.0)	1.2 (0.1 to 2.4)
Urban	24.8 (24.2 to 25.3)	1 [Reference]	23.7 (23.3 to 24.0)	1 [Reference]
Insurance type				
Private	20.3 (19.9 to 20.6)	1 [Reference]	19.8 (19.6 to 20.0)	1 [Reference]
Public	28.2 (27.6 to 28.8)	7.9 (7.2 to 8.7)	28.2 (27.8 to 28.7)	8.4 (7.9 to 8.9)
Other	24.3 (22.4 to 26.2)	4.0 (2.1 to 6.0)	22.7 (22.1 to 23.3)	2.9 (2.3 to 3.5)
None	41.2 (40.4 to 42.0)	20.9 (20.1 to 21.8)	40.9 (40.3 to 41.6)	21.1 (20.5 to 21.8)

Abbreviation: LGBQ+, lesbian, gay, bisexual, other, and unsure.

^a^ Based on data from the National Health Interview Survey.

^b^ Model was simultaneously adjusted for variables shown in the table.

^c^ Differences in predicted probability reflect the risk relative to the reference group, adjusted for variables in the model.

^d^ Other category includes Alaska Native, American Indian, and other single and multiple races or ethnicities.

^e^ All sexual orientations other than heterosexual (gay, lesbian, bisexual, other sexual orientation, and/or unsure about sexual orientation) were combined into a single LGBQ+ category because of the small sample size; transgender individuals could not be identified in the data, which include only a binary sex variable defined as male or female; hence the use of the term *LGBQ+*.

## Discussion

This cross-sectional study analyzed the rates of overdue cervical cancer screening and the reasons for not receiving screening by age, race and ethnicity, sexual orientation, rurality of residence, and insurance type among a nationally representative sample of women in the US. We also identified the change in rates of overdue screening and the reasons for not receiving timely screening over a 14-year period. On a national level, the proportion of women without up-to-date screening increased from 14.4% in 2005 to 23.0% in 2019.

The proportion of women reporting lack of access as their primary barrier to receiving up-to-date screening decreased significantly from 2005 to 2019 across all age groups, possibly representing benefits from Medicaid expansion,^15,16^ the Affordable Care Act requirement that USPSTF-recommended preventive care be covered by private insurance without a copayment, and other Affordable Care Act provisions, such as the extension of dependent coverage to young adults and coverage through state exchanges.^17^ Those who reported not being up to date on screening because they received the HPV vaccine represented only 1% of women aged 21 to 29 years in 2019. Human papillomavirus vaccination was even less likely to be a reason for not being up to date on screening among those aged 30 to 65 years because most women in that age group were not generally eligible to receive the HPV vaccine, and coverage among those who were eligible to receive the vaccine was fairly low. In addition, a previous study found that women aged 21 to 39 years who did not receive an HPV vaccine were significantly more likely to be overdue for cervical cancer screening compared with those who received an HPV vaccine, further increasing their risk of cervical cancer.^18^

The primary reason for not having up-to-date screening among all groups in all years was not knowing that screening was needed. The fact that this reason increased over time across most sociodemographic groups suggests a need for interventions targeting screening awareness for all women. Among women in the older age group, those responding that they did not receive screening because they did not have a recommendation from their health care practitioners doubled in 2019, from 5.5% to 12.0%. The importance of recommendations from health care professionals in promoting cervical cancer screening has been reported in previous studies^19,20^ and may play an even larger role as access to care continues to improve.

A potential mediator of the changes in both knowledge and practitioner recommendations may be the updates to cervical cancer screening guidelines that occurred over this period,^2,21^ leaving both patients and practitioners uncertain of the timing and recommended intervals for screening.^22,23^ Studies have suggested that changing guidelines may produce an increase in both overscreening and underscreening,^8,24^ but those already at higher risk of cervical cancer may be most susceptible to underscreening.^25^ Therefore, it was not unexpected to find many traditionally marginalized sociodemographic groups had the highest risk of overdue screening. Although lack of knowledge was a common barrier across all sociodemographic groups, variation was observed in both overdue screening rates and reasons for not receiving up-to-date screening by sociodemographic characteristics. For example, we found both Asian and Hispanic women were more likely to have screening that was not up to date compared with non-Hispanic White women, but the reasons varied across race and ethnicity. Although both Asian and Hispanic women reported lack of knowledge as a barrier, Asian women were more likely to report lack of recommendation from a health care professional and perception of no problems as barriers, whereas Hispanic women were twice as likely as Asian women to report lack of access as a barrier.

Women identifying as LGBQ+ were less likely than those identifying as heterosexual to report lack of knowledge as a barrier, but almost 10% of women identifying as LGBQ+ reported other reasons for not receiving timely screening, which merits future research into specific factors that may be associated with low screening rates in this population. When seeking to reduce disparities in cervical cancer outcomes, it will be important to design interventions that are flexible to targeted individuals' needs, barriers, and facilitators. Although educational and practitioner-focused interventions may improve screening for many women, others may not benefit if access barriers are not addressed.

### Limitations

This study has several limitations. Women could only endorse 1 response as the primary reason for not receiving up-to-date screening. It is likely that many women, particularly women who are systematically disadvantaged, including those without insurance, those from racial and ethnic minority groups, and those identifying as LGBQ+, may have multiple barriers to screening. Furthermore, cervical cancer screening itself is insufficient to reduce cervical cancer burden; barriers to follow-up in the event of abnormal findings are also common^26^ and may not overlap with screening barriers.^27^ Addressing both types of barriers will be important to reducing excess cervical cancer morbidity and mortality.

The NHIS assessed reasons for not receiving timely screening in 2019 but only asked for reasons among those who reported they had never received screening or had not received screening in the last 5 years. Given that a 3-year screening interval was recommended for women aged 20-29 years in 2019, our assessment of reasons for not receiving timely screening is missing data from some women aged 21 to 29 years who were overdue for screening by less than 2 years. However, this population is comparatively small (9.4% of the women aged 21-29 years who did not receive up-to-date screening in 2019) and addressing barriers among those with the longest screening delays is of higher importance.

Our measure of up-to-date screening relied on self-reporting, which has consistently been found to overreport screening prevalence, including differential overreporting by race and ethnicity.^28,29^ Using data from insurance claims or the electronic health record may provide a more sensitive and specific measure of screening use but may also miss women who are uninsured or not receiving regular health care. Furthermore, claims data are unable to measure the reasons women have not received timely screening, particularly within demographic groups, which is an important contribution of our study.

## Conclusions

This cross-sectional study found that USPSTF guideline–concordant cervical cancer screening in the US decreased between 2005 and 2019. Lack of knowledge of screening and lack of screening recommendations from health care professionals may be 2 modifiable barriers to timely cervical cancer screening. However, the findings also revealed that barriers to screening significantly varied by sociodemographic factors, suggesting cultural adaptation of interventions will be an important factor in the success of efforts to increase cervical cancer screening uptake among priority populations in the US, including women of Asian race and Hispanic ethnicity, women without insurance, women living in rural areas, and/or women identifying as LGBQ+. Along with increasing HPV vaccine coverage, improving cervical cancer screening rates represents an important strategy for national campaigns to eliminate cervical cancer as a public health concern.^30,31^

## Notes

Supplement. **eTable 1.** Proportions of Women Without Up-to-Date Screening in 2019, NHIS **eTable 2.** Proportions of Primary Reasons for Not Receiving Screening by Sociodemographic Factors in 2019, NHIS **eTable 3.** Proportions of Women Without Up-to-Date Screening by Sociodemographic Factors Over 14 Years (2005 vs 2019), NHIS **eTable 4.** Proportions of Primary Reasons for Not Receiving Screening by Sociodemographic Factors Over 14 Years (2005 vs 2019), NHIS
