# Supplementary material for: Assessment of US Preventive Services Task Force Guideline–Concordant Cervical Cancer Screening Rates and Reasons for Underscreening by Age, Race and Ethnicity, Sexual Orientation, Rurality, and Insurance, 2005 to 2019
Source: JAMA Netw Open. 2022 Jan 18;5(1):e2143582. doi: 10.1001/jamanetworkopen.2021.43582 (PMC8767443; doi:10.1001/jamanetworkopen.2021.43582)
Supplement: Supplement. — eTable 1. Proportions of Women Without Up-to-Date Screening in 2019, NHIS eTable 2. Proportions of Primary Reasons for Not Receiving Screening by Sociodemographic Factors in 2019, NHIS eTable 3. Proportions of Women Without Up-to-Date Screening by Sociodemographic Factors Over 14 Years (2005 vs 2019), NHIS eTable 4. Proportions of Primary Reasons for Not Receiving Screening by Sociodemographic Factors Over 14 Years (2005 vs 2019), NHIS [file jamanetwopen-e2143582-s001.pdf]

Supplementary Online Content

Suk R, Hong YR, Rajan SS, Xie Z, Zhu Y, Spencer JC. Assessment of US Preventive Services Task Force guideline–concordant cervical cancer screening rates and reasons for underscreening by age, race and ethnicity, sexual orientation, rurality, and insurance, 2005 to 2019. *JAMA Netw Open*. 2022;5(1):e2143582. doi:10.1001/jamanetworkopen.2021.43582

eTable 1. Proportions of Women Without Up-to-Date Screening in 2019, NHIS

eTable 2. Proportions of Primary Reasons for Not Receiving Screening by Sociodemographic Factors in 2019, NHIS

eTable 3. Proportions of Women Without Up-to-Date Screening by Sociodemographic Factors Over 14 Years (2005 vs 2019), NHIS

eTable 4. Proportions of Primary Reasons for Not Receiving Screening by Sociodemographic Factors Over 14 Years (2005 vs 2019), NHIS

This supplementary material has been provided by the authors to give readers additional information about their work.

© 2022 Suk R et al. *JAMA Network Open*.

eTable 1. Proportions of Women Without Up-to-Date Screening in 2019, NHIS

**Socio-demographic factors**	**Not up-to-date**
	n (Weighted N)<br>% (95% CI)
**Age**	
**21-29**	481 (5,538,625)<br>29.1% (26.4-31.9)
**30-65**	1,568 (12,717,655)<br>21.1% (19.9-22.3)
p-value	*<0.001*
**Race/ethnicity**	
**NH White**	1,116 (9,192,478)<br>20.1% (18.8-21.4)
**NH Black**	242 (2,230,463)<br>21.5% (18.7-24.3)
**Hispanic**	440 (4,418,102)<br>29.1% (26.2-31.9)
**Asian**	186 (1,778,983)<br>31.4% (27.0-35.8)
**Other**	65 (636,254)<br>27.1% (18.9-35.2)
p-value	*<0.001*
**Sexual orientation**	
**Heterosexual**	1,838 (16,236,284)<br>22.2% (21.1-23.4)
**LGBQ+**	185 (1,796,550)<br>32.0% (27.5-36.5)
p-value	*<0.001*
**Rurality**	
**Urban**	1,724 (15,720,572)<br>22.6% (21.4-23.8)
**Rural**	325 (2,535,709)<br>26.2% (22.8-29.6)
p-value	*0.048*
**Insurance**	
**Private**	1,087 (9,558,373)<br>18.1% ((16.9-19.3)
**Public**	369 (3,391,480)<br>27.9% (24.9-30.9)
**Other**	83 (585,221)<br>21.5% (16.2-26.8)
**Uninsured**	434 (4,291,785)<br>41.7% (38.1-45.2)
p-value	*<0.001*

Abbreviations: n, unweighted counts; N, weighted counts; CI, confidence interval; UTD, up-to-date; NH, non-Hispanic; LGBQ+, lesbian/gay, bisexual, other, and unsure.

© 2022 Suk R et al. *JAMA Network Open*.

eTable 2. Proportions of Primary Reasons for Not Receiving Screening by Sociodemographic Factors in 2019, NHIS

**Socio-demographic factors**	**Do not know**	**No recommendation**	**No problem**	**Lack of access**	**Postponed**	**Unpleasant**	**Vaccinated**	**Other**
	n (WN)<br>% (95% CI)	n (WN)<br>% (95% CI)	n (WN)<br>% (95% CI)	n (WN)<br>% (95% CI)	n (WN)<br>% (95% CI)	n (WN)<br>% (95% CI)	n (WN)<br>% (95% CI)	n (WN)<br>% (95% CI)
**Age**								
**21-29**	257 (3,013,097)<br>60.0% (54.6-65.5)	45 (550,683)<br>11.0% (7.5-14.4)	38 (390,738)<br>7.8% (4.8-10.7)	21 (283,314)<br>5.6% (2.8-8.5)	23 (268,069)<br>5.3% (3.0-7.7)	8 (124,606)<br>2.5% (0.7-4.3)	4 (49,572)<br>1.0% (0.0-2.1)	26 (339,601)<br>6.8% (3.6-9.9)
**30-65**	691 (5,874,790)<br>54.8% (51.3-58.2)	162 (1,284,613)<br>12.0% (9.9-14.0)	175 (1,410,616)<br>13.2% (10.7-15.6)	121 (1,044,209)<br>9.7% (7.9-11.6)	66 (484,485)<br>4.5% (3.3-5.7)	30(173,535)<br>1.6%(1.0-2.3)	0 (0)<br>0% (0-0)	58 (454,886)<br>4.2% (3.0-5.5)
p-value	*0.105*	*0.620*	*0.006*	*0.021*	*0.544*	*0.387*	-	*0.116*
**Race/ ethnicity**								
**NH White**	442 (3,786,340)<br>50.0% (45.7-54.4)	106 (898,551)<br>11.9% (9.2-14.5)	119 (940,638)<br>12.4% (9.7-15.2)	90 (785,328)<br>10.4% (8.0-12.8)	54 (418,046)<br>5.5% (3.9-7.2)	24 (188,395)<br>2.5%(1.3-3.7)	2 (13,031)<br>0.2%(0-0.4)	59 (535,715)<br>7.1% (5.0-9.2)
**NH Black**	128 (1,227,731)<br>63.2% (55.2-71.2)	31 (263,699)<br>13.6% (8.4-18.8)	19 (142,427)<br>7.3% (3.7-11.0)	13 (121,185)<br>6.2% (2.5-10.0)	7 (82,082)<br>4.2% (0.9-7.6)	2 (9,322)<br>0.5%(0-1.3)	0(0)<br>0% (0-0)	9 (95,320)<br>4.9% (0.7-9.1)
**Hispanic**	247 (2,605,535)<br>64.4% (58.9-69.9)	43 (395,185)<br>9.8% (6.4-13.1)	43 (404,256)<br>10.0% (6.1-13.9)	30 (342,658)<br>8.5% (5.1-11.9)	18 (135,933)<br>3.4% (1.6-5.2)	8(53,882)<br>1.3% (0.3-2.4)	1 (18,313)<br>0.5%(0-1.3)	7 (90,108)<br>2.2% (0.5-4.0)
**Asian**	101 (981791)<br>59.2% (50.0-68.4)	23 (223634)<br>13.5% (7.5-19.5)	24 (223876)<br>13.5% (6.1-20.9)	7 (53,032)<br>3.2% (0.6-5.8)	7 (98,636)<br>5.9% (1.2-10.7)	3 (39,225)<br>2.4% (0-5.4)	0(0)<br>0% (0-0)	5 (37,937)<br>2.3% (0-4,6)
**Other**	30 (286,490)<br>53.5% (35.8-71.3)	4 (54,226)<br>10.1% (2.7-17.6)	8 (90,158)<br>16.9% (2.4-31.3)	2 (25,321)<br>4.7% (0-13.0)	3 (17,857)<br>3.3% (0.2-6.5)	1 (7,318)<br>1.4%(0-4.1)	1 (18,228)<br>3.4%(0-10.1)	4 (35,407)<br>6.6% (0-14.2)
p-value	*<0.001*	*0.642*	*0.203*	*0.004*	*0.444*	*0.087*	-	*0.004*
**Sexual orientation**								
**Heterosexual**	862 (7,992,020)<br>57.5% (54.5-60.6)	182 (1,586,104)<br>11.4% (9.6-13.3)	187 (1,596,391)<br>11.5% (9.5-13.5)	128 (1,202,069)<br>8.7% (7.0-10.3)	72 (619,883)<br>4.5% (3.3-5.6)	33(258,370)<br>1.9%(1.1-2.6)	1 (3,577)<br>0% (0-0.1)	71 (629,507)<br>4.5% (3.1-6.0)
**LGBQ+**	75 (779,099)<br>47.2% (37.9-56.6)	22 (225,447)<br>13.7% (7.4-20.0)	24 (183,875)<br>11.1% (6.3-16.0)	14 (125,455)<br>7.6% (3.2-12.0)	16 (128,292)<br>7.8% (3.5-12.1)	2 (18,615)<br>1.1%(0-2.8)	3 (45,996)<br>2.8% (0-6.0)	11 (142,540)<br>8.6% (3.3-14.0)
p-value	0.035	0.492	0.895	0.658	0.145	0.444	0.098	0.145
**Rurality**								
**Urban**	807 (7,784,887)<br>57.1% (54.0-60.3)	179 (1,591,745)<br>11.7% (9.8-13.6)	170 (1,488,186)<br>10.9% (8.9-12.9)	113 (1,095,416)<br>8.0% (6.4-9.7)	78 (682,595)<br>5.0% (3.8-6.2)	32 (263,306)<br>1.9%(1.1-2.7)	4 (49,572)<br>0.4%(0-0.8)	71 (666,905)<br>4.9% (3.3-6.4)
**Rural**	141 (1,103,000)<br>51.9% (43.7-60.1)	28 (243,550)<br>11.5% (6.8-16.2)	43 (313,169)<br>14.7% (8.5-20.9)	29 (232,108)<br>10.9% (6.4-15.4)	11 (69,959)<br>3.3% (0.8-5.8)	6 (348,350)<br>1.6%(0.3-3.0)	0(0)<br>0% (0-0)	13 (127,582)<br>6.0% (2.6-9.4)

© 2022 Suk R et al. *JAMA Network Open*.

p-value	0.241	0.933	0.255	0.226	0.222	0.713	-	0.555
**Insurance**								
**Private**	510 (4,796,247) 59.2% (55.2-63.2)	123 (1,084,017) 13.4% (10.7-16.1)	112 (913,968) 11.3% (8.9-13.7)	30 (275,476) 3.4% (2.0-4.8)	56 (466,818) 5.8% (4.1-7.4)	19 (161,900) 2.0% (1.0-3.0)	3 (31,345) 0.4%(0-0.9)	42 (375,872) 4.6% (3.0-6.3)
**Public**	190 (1,786,588) 59.1% (52.8-65.3)	37 (356,299) 11.8% (7.6-16.0)	37 (298,629) 9.9% (6.0-13.7)	14 (118,266) 3.9% (1.6-6.2)	20 (191,100) 6.3% (2.9-9.7)	8 (65,915) 2.2%(0.5-3.8)	0(0) 0% (0-0)	21 (206,867) 6.8% (3.3-10.4)
**Other**	37 (306,945) 60.8% (46.9-74.8)	11 (72,008) 14.3% (5.8-22.8)	7 (33,167) 6.6% (0.8-12.3)	3 (27,131) 5.4% (0-13.1)	2 (14,295) 2.8% (0-6.9)	4 (38,332) 7.6% (0-17.1)	0(0) 0% (0-0)	2 (12,733) 2.5% (0-6.1)
**Uninsured**	181 (1,824,177) 48.6% (42.3-55.0)	24 (255,118) 6.8% (3.8-9.8)	46 (479,970) 12.8% (8.0-17.6)	94 (903,796) 24.1% (19.1-29.1)	9 (64,117) 1.7%(0.6-2.9)	3 (22,377) 0.6%(0-1.4)	1 (18,228) 0.5%(0-1.4)	15 (182,131) 4.9% (1.9-7.8)
p-value	0.038	0.010	0.439	<0.001	<0,001	0.063	-	0.4777

Abbreviations: n, unweighted sample size; WN, weighted sample size; %, weighted percentage; CI, confidence interval; NH, non-Hispanic; LGBQ+, lesbian/gay, bisexual, other, and unsure.

© 2022 Suk R et al. *JAMA Network Open*.

eTable 3. Proportions of Women Without Up-to-Date Screening by Sociodemographic Factors Over 14 Years (2005 vs 2019), NHIS

	**Year**	**Not up-to-date**
		n (WN)<br>% (95% CI)
**Overall**	2005	1,600 (4,507,734)<br>14.4% (13.7-15.2)
	2019	2,049 (18,,256,280)<br>23.0% (21.9-24.1)
	p-value	<0.001
**Age**		
**21-29**	2005	361 (1,061,016)<br>14.2% (12.6-15.9)
	2019	481 (5,538,625)<br>29.1% (26.4-31.9)
	p-value	<0.001
**30-65**	2005	1239 (3,446,718)<br>14.5% (13.6-15.3)
	2019	1,568 (12,717,655)<br>21.1% (19.9-22.3)
	p-value	<0.001
**Race/ethnicity**		
**NH White**	2005	806 (2,764,433)<br>12.7% (11.8-13.6)
	2019	1116 (9,192,478)<br>20.1% (18.8-21.4)
	p-value	<0.001
**NH Black**	2005	234 (596,100)<br>14.4% (12.5-16.3)
	2019	242 (2,230,463)<br>21.5% (18.7-24.3)
	p-value	<0.001
**Hispanic**	2005	433 (732,836)<br>19.3% (17.3-21.3)
	2019	440 (4,418,102)<br>29.1% (26.2-31.9)
	p-value	<0.001
**Asian**	2005	104 (342,460)<br>32.5% (26.6-38.3)
	2019	186 (1,778,983)<br>31.4% (27.0-35.8)
	p-value	0.776
**Other**	2005	23 (71,905)<br>13.4% (8.0-18.7)
	2019	65 (636,254)<br>27.1% (18.9-35.2)
	p-value	0.010
**Insurance**		

© 2022 Suk R et al. *JAMA Network Open*.

**Private**	2005	706 (2,200,530)<br>10.2% (9.4-11.0)
	2019	1,087 (9,558,373)<br>18.1% (16.9-19.3)
	p-value	<0.001
**Public**	2005	202 (518,657)<br>16.9 (14.5-19.3)
	2019	369 (3,391,480)<br>27.9% (24.9-30.9)
	p-value	<0.001
**Other**	2005	48 (137,016)<br>11.3% (8.1-14.4)
	2019	83 (585,221)<br>21.5% (16.2-26.8)
	p-value	0.002
**Uninsured**	2005	637 (1,633,046)<br>30.9% (28.6-33.1)
	2019	434 (4,291,785)<br>41.7% (38.1-45.2)
	p-value	<0.001

Abbreviations: UTD, up-to-date; n, unweighted sample size; WN, weighted sample size; %, weighted percentage; CI, confidence interval; NH, non-Hispanic; LGBQ+, lesbian/gay, bisexual, other, and unsure.

© 2022 Suk R et al. *JAMA Network Open*.

eTable 4. Proportions of Primary Reasons for Not Receiving Screening by Sociodemographic Factors Over 14 Years (2005 vs 2019), NHIS

	Year	Do not know	No recommendation	No problem	Lack of access	Postponed	Unpleasant	Vaccina-ted	Other
		n (WN)<br>% (95% CI)	n (WN)<br>% (95% CI)	n (WN)<br>% (95% CI)	n (WN)<br>% (95% CI)	n (WN)<br>% (95% CI)	n (WN)<br>% (95% CI)	n (WN)<br>% (95% CI)	n (WN)<br>% (95% CI)
**Age**									
**21-29**	2005	198 (586,249)<br>55.3% (48.8-61.7)	37 (106,349)<br>10.0% (6.3-13.8)	36 (108,407)<br>10.2% (6.7-13.8)	62 (183,800)<br>17.3% (12.6-22.0)	12 (30,721)<br>2.9% (1.6-4,2)	8 (21,344)<br>2.0% (0.3-3.7)	NA	8 (24,146)<br>2.3% (0.5-4.1)
	2019	257 (3,013,097)<br>60.0% (54.6-65.5)	45 (550,683)<br>11.0% (7.5-14.4)	38 (390,738)<br>7.8% (4.8-10.7)	21 (283,314)<br>5.6% (2.8-8.5)	23 (268,069)<br>5.3 (3.0-7.6)	8 (124,606)<br>2.5% (0.7-4.3)	4 (49,572)<br>1.0%(0-2.1)	26 (339,601)<br>6.8% (3.6-9.9)
	p-value	*0.273*	*0.715*	*0.302*	*<0.001*	*0.075*	*0.712*	*NA*	*0.015*
**30-65**	2005	563 (1,554,490)<br>45.2% (42.3-48.1)	71 (201,878)<br>5.9% (4.5-7.2)	124 (345,042)<br>10.0% (8.2-11.9)	271 (750,277)<br>21.8% (19.2-24.3)	115 (333,143)<br>9.7% (7.9-11.5)	39 (107,872)<br>3.1% (2.2-4.1)	NA	55 (149,589)<br>4.3% (3.1-5.6)
	2019	691 (5,874,790)<br>54.8% (51.4-58.2)	162 (1,284,613)<br>12.0% (10.0-14.0)	175 (1,410,616)<br>13.2% (10.7-15.6)	121 (1,044,209)<br>9.7% (7.9-11.6)	66 (484,485)<br>4.5% (3.3-5.7)	30(173,535)<br>1.6%(1.0-2.3)	0(0)<br>0% (0-0)	58 (454,886)<br>4.2% (3.0-5.5)
	p-value	*<,0.001*	*<0.001*	*0.045*	*<0.001*	*<0.001*	*0.009*	*NA*	*0.906*
**Race/ ethnicity**									
**NH White**	2005	324 (1,173,231)<br>42.4% (38.6-46.2)	52 (179,404)<br>6.5% (4.8-8.2)	89 (281,719)<br>10.2% (7.7-12.7)	192 (645,574)<br>23.4% (20.2-26.5)	87 (277,306)<br>10.0% (7.9-12.2)	25 (87,030)<br>3.1% (2.0-4.3)	NA	37 (120,169)<br>4.3% (2.9-5.8)
	2019	442 (3,786,340)<br>50.0% (45.7-54.4)	106 (898,551)<br>11.9% (9.2-14.5)	119 (940,638)<br>12.4% (9.7-15.2)	90 (785,328)<br>10.4% (8.0-12.8)	54 (418,046)<br>5.5% (3.9-7.2)	24 (188,395)<br>2.5% (1.3-3.7)	2(13,031)<br>0.2%(0-0.4)	59 (535,715)<br>7.1% (5.0-9.2)
	p-value	0.010	<0.001	0.235	<0.001	0.001	0.421	*NA*	0.037
**NH Black**	2005	114 (289,940)<br>49.0% (42.7-55.4)	18 (54,529)<br>9.2% (5.1-13.4)	34 (82,202)<br>13.9% (10.2-17.6)	40 (97,999)<br>16.6% (12.0-21.1)	16 (35,634)<br>6.0% (3.4-8.7)	6 (12,763)<br>2.2% (0.7-3.6)	NA	5 (18,606)<br>3.1% (0.2-6.1)
	2019	128 (1,227,731)<br>63.2% (55.2-71.2)	31 (263,699)<br>13.6% (8.4-18.8)	19 (142,427)<br>7.3% (3.7-11.0)	13 (121,185)<br>6.2% (2.5-10.0)	7 (82,082)<br>4.2 (0.9-7.6)	2 (9,322)<br>0.5%(0-1.3)	0(0)<br>0% (0-0)	9(95,320)<br>4.9%(0.7-9.1)
	p-value	0.008	0.198	0.013	<0.001	0.407	0.041	*NA*	0.501
**Hispanic**	2005	248 (427,176)<br>58.3% (52.6-64.0)	28 (44,035)<br>6.0% (3.2-8.8)	21 (36,890)<br>5.0% (3.0-7.1)	86 (143,492)<br>19.6% (15.1-24.0)	17 (29,459)<br>4.0% (2.1-6.0)	15 (26,523)<br>3.6% (1.6-5.6)	NA	18 (25,261)<br>3.4% (1.8-5.1)
	2019	247 (2,605,535)<br>64.4% (58.9-69.9)	43 (395,185)<br>9.8% (6.4-13.1)	43 (404,256)<br>10.0% (6.1-13.8)	30 (342,658)<br>8.5% (5.1-11.9)	18 (135,933)<br>3.4% (1.6-5.2)	8 (53,882)<br>1.3% (0.3-2.3)	1(18,313)<br>0.5%(0-1.3)	7 (90,108)<br>2.2% (0.5-4.0)
	p-value	0.134	0.092	0.028	<0.001	0.626	0.046	*NA*	0.302
**Asian**	2005	66 (218,754)<br>63.9% (54.0-73.7)	7 (23,051)<br>6.7% (2.0-11.4)	12 (40,315)<br>11.8% (4.8-18.7)	10 (33,540)<br>9.8% (3.6-16.0)	7 (21,465)<br>6.3% (1.3-11.3)	0(0)<br>0% (0-0)	NA	2 (5,335)<br>1.0%(0-3.8)
	2019	101 (981,791)<br>59.2% (50.0-68.4)	23 (223,634)<br>13.5% (7.5-19.5)	24 (223,876)<br>13.5% (6.1-20.9)	7 (53,032)<br>3.2% (0.6-5.8)	7 (98,636)<br>5.9% (1.2-10.7)	3 (39,225)<br>2.4% (0-5.4)	0(0)<br>0% (0-0)	5 (37,937)<br>2.3% (0-4.6)
	p-value	0.50	0.091	0.738	0.061	0.928	-	*NA*	0.656

© 2022 Suk R et al. *JAMA Network Open*.

**Other**	2005	9(31,638)<br>44.0% (22.7-65.3)	3 (7,208)<br>10.0% (0-21.3)	4 (12,323)<br>17.1% (3.7-30.6)	5 (13,472)<br>18.7% (3.1-34.4)	0(0)<br>0% (0-0)	1 (2,900)<br>4.0% (0-12.1)	NA	1 (4,364)<br>6.1% (0-17.5)
	2019	30 (286,490)<br>53.5% (35.8-71.3)	4 (54,226)<br>10.1% (2.7-17.6)	8 (90,158)<br>16.9% (2.4-31.3)	2 (25,321)<br>4.7% (0-12.9)	3 (17,857)<br>3.3% (0.2-6.5)	1 (7,318)<br>1.4%(0-4.1)	1(18,228)<br>3.4%(0-10.1)	4 (35,407)<br>6.6% (0-14.1)
	p-value	0.491	0.987	0.977	0.134	-	0.535	*NA*	0.937
**Insurance**									
**Private**	2005	360 (1,146,925)<br>52.2% (48.0-56.5)	58 (191,430)<br>8.7% (6.3-11.1)	85 (249,720)<br>11.4% (8.7-14.1)	60 (186,853)<br>8.5% (6.1-10.9)	83 (252,468)<br>11.5% (9.2-13.8)	28 (82,488)<br>3.8% (2.2-5.3)	NA	30(86,219)<br>3.9% (2.5-5.4)
	2019	510 (4,796,247)<br>59.2% (55.2-63.2)	123 (1,084,017)<br>13.4% (10.7-16.0)	112 (913,968)<br>11.3% (8.9-13.7)	30 (275,476)<br>3.4% (2.0-4.8)	56 (466,818)<br>5.8% (4.1-7.4)	19 (161,900)<br>2.0% (1.0-3.0)	3(31,345)<br>0.4%(0-0.9)	42 (375,872)<br>4.6% (3.0-6.3)
	p-value	0.020	0.011	0.959	<0.001	<0.001	0.058	*NA*	0.521
**Public**	2005	115 (297,012)<br>57.3% (50.0-64.5)	18 (53,329)<br>10.3% (5.9-14.6)	18 (49,937)<br>9.6% (5.7-13.5)	17 (43,904)<br>8.5% (4.6-12.3)	14 (31,902)<br>6.2% (2.8-9.5)	8 (17,140)<br>3.3% (1.2-5.4)	NA	12 (25,433)<br>4.9% (2.0-7.8)
	2019	190 (1,786,588)<br>59.1% (52.8-65.3)	37 (356,299)<br>11.8% (7.6-16.0)	37 (298,629)<br>9.9% (6.0-13.7)	14 (118,266)<br>3.9% (1.6-6.2)	20 (191,100)<br>6.3% (2.9-9.7)	8 (65,915)<br>2.2% (0.5-3.8)	0 (0)<br>0% (0-0)	21 (206,867)<br>6.8% (3.3-10.4)
	p-value	0.707	0.631	0.930	0.048	0.944	0.402	*NA*	0.408
**Other**	2005	28 (80,376)<br>58.7% (45.7-71.6)	5 (13,070)<br>9.5% (1.2-17.8)	5 (13,713)<br>10.0% (1.5-18.5)	6 (20,681)<br>15.1% (7.9-22.3)	4 (9,176)<br>6.7% (0.7-12.7)	0(0)<br>0% (0-0)	NA	0(0)<br>0% (0-0)
	2019	37 (306,945)<br>60.8% (46.9-74.8)	11 (72,008)<br>14.3% (5.8-22.7)	7 (33,167)<br>6.6% (0.8-12.3)	3 (27,131)<br>5.4% (0-13.0)	2 (14,295)<br>2.8% (0-6.9)	4 (38,332)<br>7.6% (0-17.1)	0 (0)<br>0% (0-0)	2 (12,733)<br>2.5% (0-6.1)
	p-value	0.822	0.422	0.515	0.066	0.293	-	*NA*	-
**Uninsured**	2005	254 (606,112)<br>37.1% (33.1-41.1)	26 (49,286)<br>3.0% (1.4-4.7)	51 (136,749)<br>8.4% (6.0-10.8)	250 (682,639)<br>41.8% (37.3-46.3)	25 (66,589)<br>4.1% (2.4-5.7)	11((29,588)<br>1.8%(0.5-3.1)	NA	20 (62,083)<br>3.8% (1.9-5.7)
	2019	181 (1,824,177)<br>48.6% (42.3-55.0)	24 (255,118)<br>6.8% (3.8-9.8)	46 (479,970)<br>12.8% (8.0-17.6)	94 (903,796)<br>24.1% (19.1-29.1)	9 (64,117)<br>1.7%(0.6-2.9)	3 (22,377)<br>0.6%(0-1.4)	1 (18,228)<br>0.5% (0-1.4)	15 (182,131)<br>4.9% (1.9-7.8)
	p-value	0.003	0.030	0.109	<0.001	0.023	0.109	*NA*	0.554

Abbreviations: n, unweighted sample size; WN, weighted sample size; %, weighted percentage; CI, confidence interval; NH, non-Hispanic; LGBQ+, lesbian/gay, bisexual, other, and unsure.

© 2022 Suk R et al. *JAMA Network Open*.
